# Towards water-soluble [60]fullerenes for the delivery of siRNA in a prostate cancer model

**DOI:** 10.1038/s41598-021-89943-5

**Published:** 2021-05-19

**Authors:** Julia Korzuch, Monika Rak, Katarzyna Balin, Maciej Zubko, Olga Głowacka, Mateusz Dulski, Robert Musioł, Zbigniew Madeja, Maciej Serda

**Affiliations:** 1 Institute of Chemistry, University of Silesia in Katowice, 40-006 Katowice, Poland; 2 Faculty of Biochemistry, Biophysics and Biotechnology, Jagiellonian University, 30-387 Kraków, Poland; 3 Institute of Physics and Silesian Center for Education and Interdisciplinary Research, University of Silesia in Katowice, 41-500 Chorzów, Poland; 4 Institute of Materials Engineering, University of Silesia in Katowice, 41-500 Chorzów, Poland; 5 Department of Physics, Faculty of Science, University of Hradec Králové, 500-03 Hradec Králové, Czech Republic

**Keywords:** Chemical biology, Medicinal chemistry, Cancer, Chemical biology, Drug discovery, Chemistry, Nanoscience and technology, Nanomedicine

## Abstract

This paper presents two water-soluble fullerene nanomaterials (HexakisaminoC_60_ and monoglucosamineC_60_, which is called here JK39) that were developed and synthesized as non-viral siRNA transfection nanosystems. The developed two-step Bingel–Hirsch reaction enables the chemical modification of the fullerene scaffold with the desired bioactive fragments such as d-glucosamine while keeping the crucial positive charged ethylenediamine based malonate. The ESI–MS and ^13^C-NMR analyses of JK39 confirmed its high T_*h*_ symmetry, while X-ray photoelectron spectroscopy revealed the presence of nitrogen and oxygen-containing C–O or C–N bonds. The efficiency of both fullerenes as siRNA vehicles was tested in vitro using the prostate cancer cell line DU145 expressing the GFP protein. The HexakisaminoC_60_ fullerene was an efficient siRNA transfection agent, and decreased the GFP fluorescence signal significantly in the DU145 cells. Surprisingly, the glycofullerene JK39 was inactive in the transfection experiments, probably due to its high zeta potential and the formation of an extremely stable complex with siRNA.

## Introduction

Nanotechnology has changed traditional medicinal chemistry, by enabling the development of small molecular drugs with targeted nanotherapeutics^1^. The central premise of medical chemistry is that a specific small molecule can bind to the desired enzyme or receptor, which results in a specific therapeutic effect. The aforementioned inhibition process can also be achieved using nucleic acid therapies including a technique called RNA interference (Nobel Prize 2006) in the presence of engineered nanomaterials used as transfection agents^2,3^. An efficient transfection agent must deliver the targeted siRNA into the cytoplasm where it degrades the targeted mRNA after binding to the argonaute proteins (AGO) and the further formation of the RNA-induced silencing complex (RISC)^4^. Recently, the FDA has approved an siRNA-based drug (lipid nanoparticles, patisiran) to treat transthyretin-mediated amyloidosis^5^. Due to increased interest in the nanomedical approaches in molecular biology, the interactions of nucleic acids and carbon nanomaterials have been thoroughly studied and discussed, mainly focusing on the interactions of the DNA-carbon nanotubes/cationic fullerenes^6,7^. The use of [60]fullerene hexakisadducts as DNA transfection agents was previously described in case of multivalent cationic fullerenes and polycationic fullerenes, which formed a stable complexes with DNA plasmids with minimal cytotoxicity in mammalian cells^8–10^. Although the interaction of the cationic fullerenes with DNA is well presented even in murine models, the siRNA transfection techniques have mainly been developed for only one derivative, TPFE (*tetra*-piperazino-[60]fullerene epoxide)^11^, which forms a stable 7 nm micelle, but has limited options for its further chemical functionalization or the addition of targeting groups or ligands for biorthogonal/click chemistry. Furthermore, the main advantages of fullerene-based transfection agents over cationic lipids are the high water-solubility, the ability to cross biological membranes, low cytotoxicity and a high synthetic accessibility allowing additional options for bioconjugation with desired drugs in engineered nanomaterials. Moreover, it has been proven that TPFE is non-toxic and is effective for lung-targeted in vivo siRNA delivery, which is based on the formation of the micrometer-sized TPFE–siRNA–serum protein complexes, which could be a stabilization factor for relatively unstable siRNA under physiological conditions^12^. Studies performed by Wang et al. described a fullerene-ethylenediamine modified dextran hybrid (C_60_-Dex-NH_2_) as an efficient siRNA transfection agent when they evaluated it in the human breast cancer cell line MDA-MB-231, which could be photo-activated and could destroy the endo-lysosomal membranes via a controllable generation of ROS^13^. Simultaneously, the interactions of carbon nanomaterials with biological fluids are crucial factors that determine their cellular fate and further tissue targeting^14,15^. Experimental and computational studies have been performed to describe the formation of complexes between the water-soluble carboxylated fullerenes and serum albumins and other proteins such as lysosome and the serine proteinases^16,17^. Our previous studies showed that the protein corona is formed on the surface of glycofullerenes and modulates their inhibitory activity, however its detailed composition is still unknown^18^. Due to nanoparticle zeta-potentials influence on the formation and composition of the protein corona, it was observed that in positively charged nanoparticles after their complexation with the plasma proteins, the complex's final zeta potential decreases^19^.

Herein a facile methodology for the synthesis of highly water-soluble [60]fullerene hexakisadducts is presented using the Bingel-Hirsch synthetic approach to modify the buckyball scaffold. Our robust protocol was used to create two highly functionalized *T*_*h*_ symmetrical fullerene nanomaterials including monoglucosamineC_60_ which is called here a JK39 compound with a d-glucosamine’s fragment. The rationale behind this approach was to increase the interactions of the nucleic acids with a sugar fragment as has been described for the chitosan-based transfection agents^20^. The additional advantage of fullerene hexakisadducts is that they limit the occurrence of several regioisomers, simplify of the purification process and increase water-solubility in contrast to the fullerene monoadducts, which is particularly useful in biological experiments^21,22^. During the designing step, we kept in mind that the glycofullerenes that we previously reported on, could localize in the nuclear envelope- which could be a beneficial property for the transfection process^23^. We previously described HexakisaminoC_60_ as being a non-toxic photosensitizer that could be used to generate reactive oxygen species as described for the treatment non-melanoma skin cancer^24^. The engineered water-soluble aminofullerenes HexakisaminoC_60_ and JK39 were designed as dual-acting nanotherapeutics, which degrade a targeted mRNA in a sequence-specific manner that might have a potential additional photodynamic activity. Considering the interactions of fullerenes with serum albumins, the transfection experiments were conducted in a FBS-containing medium to test the ability of the synthesized carbon nanomaterials to transfect the desired siRNA. We selected Lipofectamine 3000 because it is a modern transfection agent that is commonly used in molecular biological experiments^25^.

## Methods

### Materials

All of the compounds that were used reagent grade or better, and the solvents were used as they were received unless otherwise specified. The following reagents were used as received: C_60_ (99.5 + %, SES Research, U.S.A.), d-glucosamine hydrochloride (Sigma Aldrich), DBU (1,8-diaza-bicyclo[5.4.0]undec-7-ene, Sigma Aldrich), ethyl hydrogen malonate (Sigma Aldrich), CBr_4_ (Sigma Aldrich) and DIC. The following reagents: acetic anhydride (Fisher), pyridine (Sigma Aldrich), and DMF (Sigma Aldrich) were prepared according to the procedures in the literature, distilling them with calcium hydride and were then used immediately. The Lipofectamine 3000, which was used as the control siRNA transfection agent, was obtained from Thermo Fisher Scientific (U.S.A.). For the in vitro experiments we have used the human prostate cancer (DU145) cell line (American Type Culture Collection, Rockville, U.S.A.), 24-well plates (Corning; Falcon^®^), a DMEM F12 Ham medium (Sigma-Aldrich, St.Louis, MO, USA), fetal bovine serum (FBS; Gibco), Penicillin–Streptomycin (Sigma-Aldrich, St.Louis, MO, USA), PTAI-11 (trimethyl undecaprenyl ammonium iodide; Collection of Polyprenols, Institute of Biochemistry and Biophysics PAS, Warsaw, Poland; PTAI-11 is patented (No. 231158, Polish Patent Office 2019; No. 230096, Polish Patent Office 2018, No. 211824, Polish Patent Office 2012) and there is a patent application pending (No. PCT/PL2015/000093, WO/2016/032348, Polish Patent Office, European Patent Office), DOPE (1,2-dioleoyl-*sn*-glycero-3-phosphoethanolamine; Sigma-Aldrich, St.Louis, MO, USA), DC-cholesterol (3ß-[*N*-(*N*',*N*'-dimethylaminoethane)-carbamoyl]cholesterol hydrochloride; Sigma-Aldrich, St.Louis, MO, USA).

The nuclear magnetic resonance spectra were measured on a *Bruker Avance III 500 MHz NMR Spectrometer* with TMS as the internal standard. The MS spectra were collected using an electrospray ionization time-of-flight (ESI-microTOF) mass spectrometer from Bruker Daltonics Inc (U.S.A.). The high-resolution mass spectrometry was carried out on the ESI-Q-TOF maXis impact (Bruker Daltonics Inc, U.S.A.). The purity of all the compounds was assessed using an Agilent1260 HPLC equipped with a DAAD detector at 260 nm, RP-column: Eclipse plus C_18_ (3.5 μm); flow 0.5 mL/min. The Fourier transform infrared (FTIR) measurements were taken using an Agilent Cary 640 FTIR spectrometer, that was equipped with a common source and a DTGS Peltier-cooled detector. The fullerene powders were measured using ATR diamond accessory in the 400–4000 cm^−1^ range. The spectrum was recorded at 32 accumulations and at a spectral resolution of 4 cm^−1^. The dynamic light scattering and zeta potentials for the fullerene nanomaterials and their complexes with siRNA were measured using Zetasizer Nano (Malvern Panalytical Ltd, UK). The transmission electron microscopy (TEM) observations were performed using a JEOL high resolution (HR-TEM) JEM 3010 microscope operating at a 300 kV accelerating voltage, that was equipped with a Gatan 2 k × 2 k Orius™ 833SC200D CCD camera. The chemical analysis of the surface of fullerenes was performed using the X-ray photoelectron spectroscopy (XPS) technique. The X-ray Photoelectron Spectroscopy measurements were taken using a Physical Electronic XPS spectrometer (Physical Electronics PHI 5700, Chanhassen, MN, U.S.A.). Monochromatic Al Kα radiation (1486 eV) was used to excite the photoelectrons from the surface of fullerenes. The photoemission spectra were collected in a wide binding energy range (− 2 to 1400 eV) and in the characteristic photoemission lines binding energy ranges of carbon, oxygen, nitrogen, and fluorine which was detected on samples’ surface. The analysis was carried out using PHI MultiPak (v.9.6.0.1, ULVAC PHI, Chigasaki, Japan) software. The EGFP silencing efficiency was evaluated with an OLYMPUS IX81 (Olympus) fluorescence microscope and a Guava^®^ easyCyte 8 flow cytometer (Luminex, Austin, TX, USA), at a 488 nm laser excitation. The percentage of GFP-positive cells was analyzed using InCyte software ver. 3.3 (Luminex, Austin, TX, USA).

### Synthesis of fullerene JK39 and malonic acid ligands for Bingel–Hirsch reaction

Our group previously described the HexakisaminoC_60_ synthetic protocol^24^. The robust approach for obtaining fullerene nanomaterial JK39 is depicted in Scheme S1 in the Supporting Information, and includes additional spectroscopic data. The synthesis of the [60]fullerene monoadduct (**5**), as well as its malonic acid precursor that contains the d-Glucosamine unit (4), were also previously described by our group^23^.

Ethylenediamine (15 mL; 0.25 mol) was dissolved in chloroform and cooled to 0 °C followed by the dropwise addition of 125 mL of a chloroform solution of di-*tert*-butyl bicarbonate (5.46 g; 0.025 mol) over three hours. Then, the reaction mixture was allowed to reach room temperature after which it was stirred for an additional 16 h. After that time, the solution was washed six times, with 200 mL of DI water and four times with 200 mL of brine. The organic phases were dried over MgSO_4_ and evaporated under reduced pressure. The final product was obtained as a colorless oil of *N*-Boc-protected ethylenediamine (**1**) with a 45% yield. The *N*-Boc-1,2-diaminoethane (1 g; 6.2 mmol) was dissolved in 50 mL of methanol, and then dimethylmalonate (0.39 g; 2.95 mmol) was added dropwise. The reaction mixture was refluxed for three hours and then stirred for an additional 72 h at room temperature. After that time, the methanol was evaporated in vacuo. The final product was purified by the initial extraction (DCM/H_2_O) followed by column chromatography (DCM:MeOH, 20:1). The final product (**2**) was obtained as a sticky off-white gum with13% yield.

The glycofullerene monoadduct (**5**) (45.2 mg; 0.0365 mmol) was dissolved in 10 mL of dichloromethane and 100 mL of toluene. The mixture was stirring for 15 min. Then the *N*-Boc-diethylamine malonate (142 mg; 0.365 mmol) and CBr_4_ (241.6 mg; 0.73 mmol) were dissolved in 10 mL of DCM that had been added to the fullerene solution. In the next step, DBU (66.7 mg; 0.438 mmol) was dissolved in 3 mL of dichloromethane, and 0.5 mL of the base was added to the solution for six hours. After all of the DBU was added, the reaction mixture was stirred at room temperature, for an additional 48 h, during which the color of the solution changed from red-brown to orange. The product was purified using a flash column in the gradient conditions (DCM, DCM: MeOH 10:1 to 5:1). The C_60_ hexakisadduct (**6**) structure which had a T_*h*_ symmetry was confirmed using ^13^C-NMR spectroscopy and mass spectrometry. To obtain a water-soluble fullerene derivative (**7**), the hexakisadduct was dissolved in 10 mL of DCM, and 20% trifluoroacetic acid was added. The reaction was stirred at room temperature for 10 days, during which two phases appeared in the solution. The water phase was collected, evaporated in vacuo and purified on centrifugal membranes using a 1 kDa filter membrane (Pall Corporation, U.S.A.). The top layer of the membrane was washed five times, with 15 mL of distilled water. Subsequently, the [60]fullerene nanomaterial was frozen at − 20 °C and freeze-dried. The final product was obtained as a brown solid with a 30% yield and was stored in a laboratory freezer at − 20 °C. The hexakisadduct (**7**) was characterized using NMR spectroscopy and infrared spectroscopy, and the structure was confirmed using ESI mass spectrometry.

### Imaging the siRNA-aminofullerene complexes using transmission electron microscopy

The TEM measurements that are presented in Figs. 3A,B and S8 were obtained using a JEOL high resolution (HR-TEM) JEM 3010 microscope operating at a 300 kV accelerating voltage. The samples of the fullerene–siRNA complexes (20 µL of a desired solutions in nuclease free water, R = 70; 0.45 µg siRNA GFP and 37.5 µg of fullerene nanomaterial) were deposited on a copper grid with a holey carbon amorphous film under air and then dried at room temperature for 24 h.

### siRNA transfection using the fullerene nanomaterials

The human prostate cancer (DU145) cell line was obtained from the American Type Culture Collection (Rockville, U.S.A.), nr HTB-81. The DU145 cells were seeded into the wells of a 24-well plate at a density of 5 × 10^4^ and cultivated for 24 h in a DMEM F12 Ham medium with 10% FBS without antibiotics. The [60]fullerene nanomaterials and siRNA were suspended in nuclease-free water at pH 7. Next, they were mixed at a 1:1 (*v:v*) ratio and incubated for 30 min at room temperature. After incubation, the mixture was added to the wells with the cells that contained the FBS-supplemented medium (final FBS concentration—5%). A 0.45 µg/well of siRNA GFP (Sigma Aldrich) was used with 37.5 µg of HeksakisaminoC_60_, 37.5 µg of JK39, or 1.5 µL of Lipofectamine^®^ 3000. After five hour incubation 300 µL of the medium that had been supplemented with 20% of FBS and antibiotics (Penicillin–Streptomycin [200U–0.2 mg/mL]). The mixture of PTAI-11 (trimethylundekaprenylammonium iodide; Collection of Polyprenols, Institute of Biochemistry and Biophysics PAS, Warsaw, Poland) + DOPE (1,2-dioleoyl-*sn*-glycero-3-phosphoethanolamine; Sigma Aldrich) + DC-cholesterol (3ß-[*N*-(*N*′,*N*′-dimethylaminoethane)-carbamoyl]cholesterol hydrochloride; Sigma Aldrich) at a 1:1:1 molar ratio in 3 µg of lipids was used 24 h after the siRNA had been introduced into the transfected cells with 2.7 µg of pEGFP-C1 plasmid as was previously described^26,27^. To summarize, the experimental steps were: day 1—plating cells; day 2—transfection with siRNA using fullerenes or Lipofectamine^®^ 3000; day 3—transfection with the pEGFP-C1 plasmid using PTAI-11 + DOPE + DC-cholesterol and day 4—evaluating the efficiency of EGFP silencing.

## Results and discussion

The procedure for synthesizing HexakisaminoC_60_ as well as its biophysical properties were published earlier, when investigating its photodynamic activity in the non-melanoma skin cancer model^24^. The fullerene nanomaterial JK39 was obtained in a time-controlled two-step Bingel–Hirsch reaction (for the synthetic protocol, see the Supporting Information Scheme S1), in which a peracyleted d-glucosamine fullerene monoadduct (5) was further modified in a second Bingel-Hirsch reaction with Boc-protected malonate (2) its high-resolution ESI spectrum is presented in Fig. S4. In general, the Boc protection on aminomalonate was more useful in the Bingel-Hirsch reaction in terms of the general yields and purification procedure than a previously used trityl function. As a result, T_*h*_-symmetrical hexakisadduct (6) was created, whose structure was confirmed by the presence of two fullerene sp^2^ signals at *δ* = 146 and 141 ppm along with an sp^3^ signal at *δ* = 69 ppm in the ^13^C-NMR spectrum of (6) (Fig. S2). Additionally, one could also observe three characteristic signals for different types of NH groups, which are presented between 8.20 and 7.30 ppm in ^1^H-NMR spectrum (Fig. S1) of fullerene nanomaterial (6). After the hydrolysis of [60]fullerene derivative (6) a highly water-soluble fullerene nanomaterial (7) was created, and its structure was studied using ^13^C-NMR spectroscopy. The strongest signals in ^13^C-NMR of fullerene nanomaterial (7) are those connected with TFA counter anion signals (both quartets are located around 162 and 115 ppm, Fig. S3) as well as the methylene groups in ethylenediamine fragments (NH–CH_2_CH_2_NH), located at 38 and 37 ppm, which are depicted in Fig. S3. Due to the limits of our ESI–MS detector (3000 Da), we were not able to measure the molecular peak of the protected fullerene nanomaterial (6) which had a molecular mass at 3109 Da; however, it was possible to detect signals from its fragmentation (Fig. S5). The peak at 2685 Da resulted from the fragmentation of *D*-glucosamine malonate (M = 459 Da) from the parent structure with the addition of two water molecules. In contrast, the peak at 2297 Da corresponded to the fragmental structure without an acetylated d-glucosamine malonate unit, and one Boc-protected aminomalonate (M = 388 Da), which was presented as an adduct with two water molecules. In the next step in our synthetic protocol, the acetyl and Boc protection were removed from the fullerene nanomaterial (6) via a TFA hydrolysis, which was followed by further membrane dialysis, to form the final water-soluble JK39, which was further characterized using the NMR, FTIR, and XPS techniques with additional measurements of its size (DLS) and zeta potential.

The ESI-mass spectrum of a water-soluble fullerene is depicted in Fig. 2B. We were not able to directly detect its molecular peak (M = 1941 Da) due to its polycationic nature, although the spectrum did show a huge signal at 1038 Da, which corresponded to the double-charged cationic form of fullerene (7) [M + 3Na]^2+^.

The analysis of the infrared spectrum of JK39 is very similar to previously published HexakisaminoC_60_ and should be performed by considering two spectral regions, firstly (1) 2250–3800 cm^−1^ and secondly, (2) 400–1900 cm^−1^ (Fig. 2A)^24^. According to this division, for both compounds region 1 is determined by the presence of the overlapping signals that had originated from the symmetric and asymmetric stretching modes of *ν*(CH_x_), and the ammonium cations containing ν(–CH_2_NH_x_^+^) (x = 2, 3)^28^. As a result of the introduction of the d-glucosamine fragment, an additional band at 3246 cm^−1^ can be explained by the presence of amine *ν*(NH), and it is worth noting that its position is linked to the presence of intra- or intermolecular interactions with hydroxyl groups^29^. It is also interesting that the character of the band arrangement within region 1 in both compounds indicated a considerable distribution and high number of *H*-bonds with different donor–acceptor bond lengths. Unfortunately, it was not easy to interpret and analyze the signal of the hydroxyl groups due to the high complexity of the spectrum, e.g. there was an increase in the intensity of the signal of JK39 above 3300 cm^−1^, which may indicate the presence of the hydroxyl groups as a result of the introduction of the glucosamine fragment. In turn, interpreting of the bands from region 2 was much more complicated due to the higher complexity of the molecular vibration. However, two bands at around 1665 cm^−1^ and 1527 cm^−1^ referred to the asymmetric and symmetric deformation vibration of ammonium cation, while the shoulders that were observed close to those two maxima but that were located at lower wavenumbers, are related to the vibration of the ammonium cation. The literature and previously reported data for HexakisaminoC_60_ that suggested that the position of the band at 1665 cm^−1^ might also be explained by taking into account the stretching vibration of carbonyl modes due to the occurrence of the β-diketone structure^24,30^, or due to the high dynamicity of the system through the formation of hydrogen bonds which shifted the carbonyl band maximum into lower wavenumbers. In turn, the presence of a less intense band around 1800 cm^−1^ might be explained through the presence of *N*-protonated amide moiety, which is unusual due to well-known favorable *O*-protonation process. However, it was previously discussed in the literature that some amides and peptides are *N*-protonated, especially in case of strained amides, but also for peptides with electron donating groups and alfa-effects^31–33^. Other bands at the maxima around 1430, 1180, and 1120 cm^−1^ might respectively be linked to the deformational modes of the ethylenediamine groups, the rocking vibration of the ammonium cation or the skeletal vibration of the alkane chain and deformational modes of the methylene groups. The presence of sugar-based malonate was also reflected in the occurrence of low intense bands at 1369, 1285, and 1039 cm^−1^ due to the C–O–C vibration within the pyranose ring and the deformational modes of –CH_2_CH_3_ chain.

**Figure 1:**
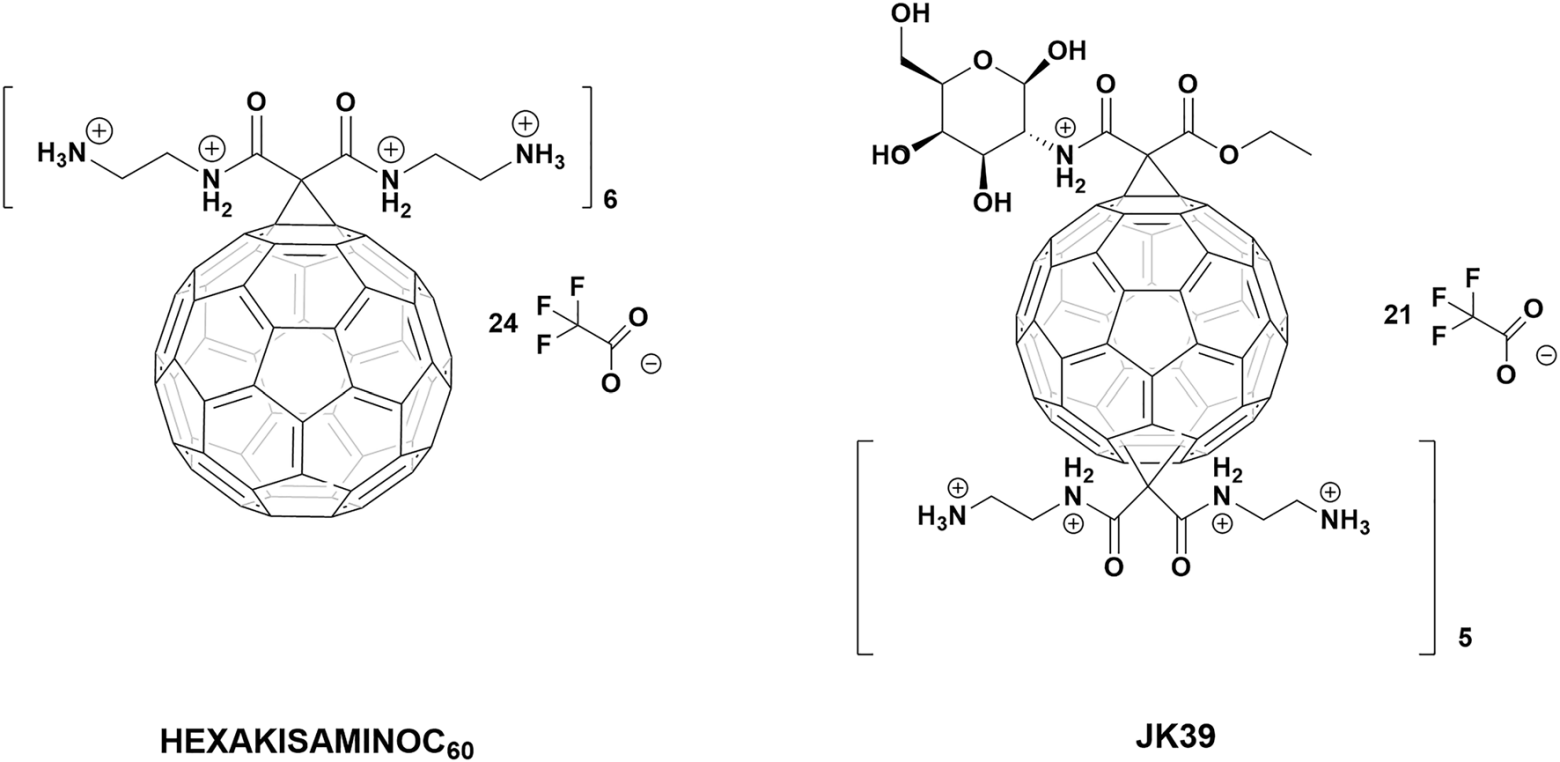
The non-viral cationic fullerene nanomaterials HexakisaminoC_60_ and JK39 that were used for the siRNA transfection.

**Figure 2:**
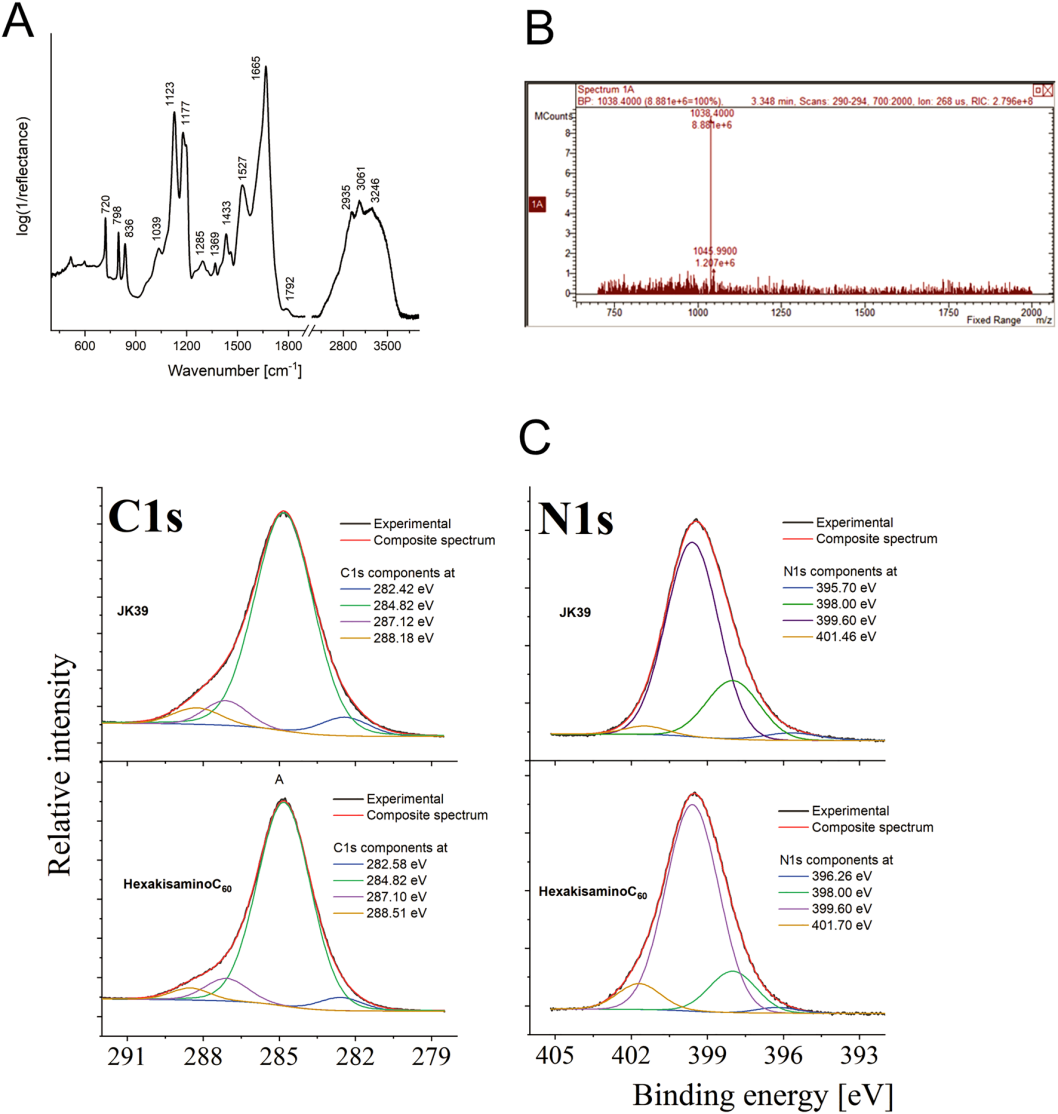
(**A**) Infrared spectrum of fullerene JK39; (**B**) ESI-mass spectrum of water-soluble fullerene JK39 (positive mode, 100 mV); (**C**) A high-resolution photoemission spectra of carbon and nitrogen measured in the fullerene nanomaterials JK39 and HexakisaminoC_60_.

The local environment detected on the fullerene surface elements was examined using the XPS technique. Based on the analysis of the XPS survey spectra, several elements were detected on the surface of both fullerene nanomaterials. The chemical composition and calculated atomic and weight concentrations are combined for both fullerenes in Table S1 (see Supporting Information). Elements with an atomic concentration below one atomic percentage, such as Si, S, Na, Cl, and Br can be treated as contaminants. An analysis of the main components of the examined samples (C, O, N, F) indicated some variations in the atomic concentration and, consequently, in the relative ratio of the individual components. The most pronounced differences are presented in the amount of the detected carbon and nitrogen as both of the fullerene nanomaterials have the same ethylenediamine core with the main difference in the d-glucosamine unit (Fig. 1). The chemical state for carbon, oxygen, nitrogen, and fluorine was determined by analyzing the high-resolution spectra of the C1s, O1s, F1s, and N1s photoemission lines. It was revealed that for a particular element, the presence of several different chemical states was related to the specific chemical bonding with the surrounding elements. The analysis of the main component of the both samples—carbon indicated that carbon existed in several different chemical states (see deconvoluted C1s line in Fig. 2C). The chemical state with the lowest binding energy (282.42 eV for sample JK39 and 282.58 eV for the HexakisaminoC_60_) was related to the occurrence of silicone contamination. The most pronounced peak in the spectra of both samples was located at 284.82 eV and was related to the presence of C–H or C–C bonds^34^. Interestingly, oxygen and nitrogen containing groups C–O or C–N (at 287.10 eV) were detected for both samples^35,36^, whereas the presence of typical carbonyl group (C=O) (288.18 eV) was only detected for fullerene JK39^37,38^. For the HexakisaminoC_60_ fullerene, the peak at 288.5 eV was assigned to the O=C–OH bond, which could be also correlated to the presence of CF_3_COO^−^ counterion^38^. The C–F (286.3 for JK39, and 286.6 eV for HexakisaminoC_60_) bond and –CF_3_ fragment of trifluoroacetic acid (687.8 eV for JK39 and 687.9 eV for HexakisaminoC_60_) were identified through the F1s deconvolution presented in Fig. S9^39^. The O1s line (Supporting Information, Fig. S9) that indicated a carbon–oxygen bond; weakly adsorbed oxygen (O_2_/OH^−^) were detected at ~ 530.3 eV, C=O (carbonyl slightly below 532 eV), and O–C=O at around 533.3 eV^40,41^. The deconvoluted N1s line (see Fig. 2C) indicated the presence of four components; the most pronounced component at 399.6 eV was assigned to the C–N^36^ or N–(C=O)– bonds^42^, the one at 398.0 eV to basic nitrogen(pyridinic type), and the chemical state at 401.4 eV to quaternary N^43^. Assuming that some of the nitrogen in the 398 eV bonding energy state could come from *N-*protonated amide fragment, the increased amount that was observed for sample JK39 (see the green line in Fig. 2C, for sample JK39, it was 22% of all of the nitrogen, for HexakisaminoC_60_—13%) could confirm the structure of sample JK39 with nitrogen attached to pyranose ring. We also calculated pKa values of all nitrogen atoms in our malonate substrates which are depicted in Fig. S10. In case of d-glucosamine containing malonate pK_a_ of the nitrogen is 19.3, while the ethylenediamine-based malonate has to pKa one for amine (10.2) and amide 19.5.

Based on our previous studies with water-soluble fullerenes and kinetic experiments on fullerene C_60_ser that were conducted Wilson’s group, we postulated that the synthesized fullerene nanomaterials would form smaller and larger aggregates in a water solution, that are in constant equilibrium^18,44^. They can disaggregate when they are exposed to a higher ionic strength (salt addition), organic solvents, and the adsorption of the protein corona on the surface of fullerene^18^. At a concentration of 0.1 mg/mL, HexakisaminoC_60_ forms aggregated at 100 nm (PDI = 0.2) with a zeta potential at + 28.6 mV^24^, while the fullerene derivative JK39 formed two subpopulations of aggregates at 134 and 599 nm (PDI = 0.365) also with two different values of the zeta potential at + 54 and + 90 mV (Figs. S6, S7, Supporting Information). The presence of two signals in the zeta potential measurement of JK39 might be caused by the considerable polydispersity of the fullerene nanomaterial and could confirm its lower stability. Additionally, the studies performed by Deryabin et al*.* on ten different fullerene derivatives demonstrated an obvious relationship between the zeta potentials of the functionalized [60]fullerene aggregates and their size in salt-free aqueous^45^. Besides, nanoparticles with a positive zeta potential have a long-circulating half-life due to the absorption of the protein corona and can form electrostatic complexes with RNA which is crucial when developing siRNA transfection agents^46^. When developing efficient fullerene nanomaterial transfection agents, certain conditions must be met. Firstly, the engineered fullerene nanomaterials should be able to form a stable complex with the desired sequence of siRNA, and that complex must deliver the RNA to the cytosol, thus protecting it from being degraded by nucleases^47^. On the other hand, the complex that is formed between the RNA and cationic fullerene should not be too stable—the desired siRNA has to cleave from the complex and perform an endosomal escape and intracellular release to form the RNA-induced silencing complex (RISC)^48^. The endosomal escape process and triggering the intracellular release tend to be the most important factors when developing effective siRNA delivery tools and are still not well understood for engineered nanomaterials^49^. Here, we decided to test the ability of our fullerene nanomaterials to silence the GFP fluorescence signal using a prostate cancer cell line (DU145) due to the simplicity of the model^26^. To better mimic in vivo conditions, we conducted all of the siRNA transfection experiments in the presence of FBS.

The R value (70) that used in the transfection experiments was calculated by dividing the nitrogen-to-phosphorus (N/P) ratio by two. Interestingly, due to the many positive charges placed at the nitrogen atoms in our two fullerene nanomaterials HexakisaminoC_60_ and JK39 (24 and 20, respectively), the R number was also high—the experimental values of the R parameter that were used for the siRNA transfection with the TPFE fullerene was between 20 and 50. Interestingly, in the transfection experiments carried out using the TPFE fullerene, it was dissolved in a potassium chloride solution (pH 2) to ensure the complete protonation of the aminofullerene core before the formation of complex with desired siRNA. In our experiments two fullerene nanomaterials were dissolved in nuclease free water (pH 7) without adding any buffers—the low pH of that solution could have cytotoxic effects on the cells.

**Figure 3:**
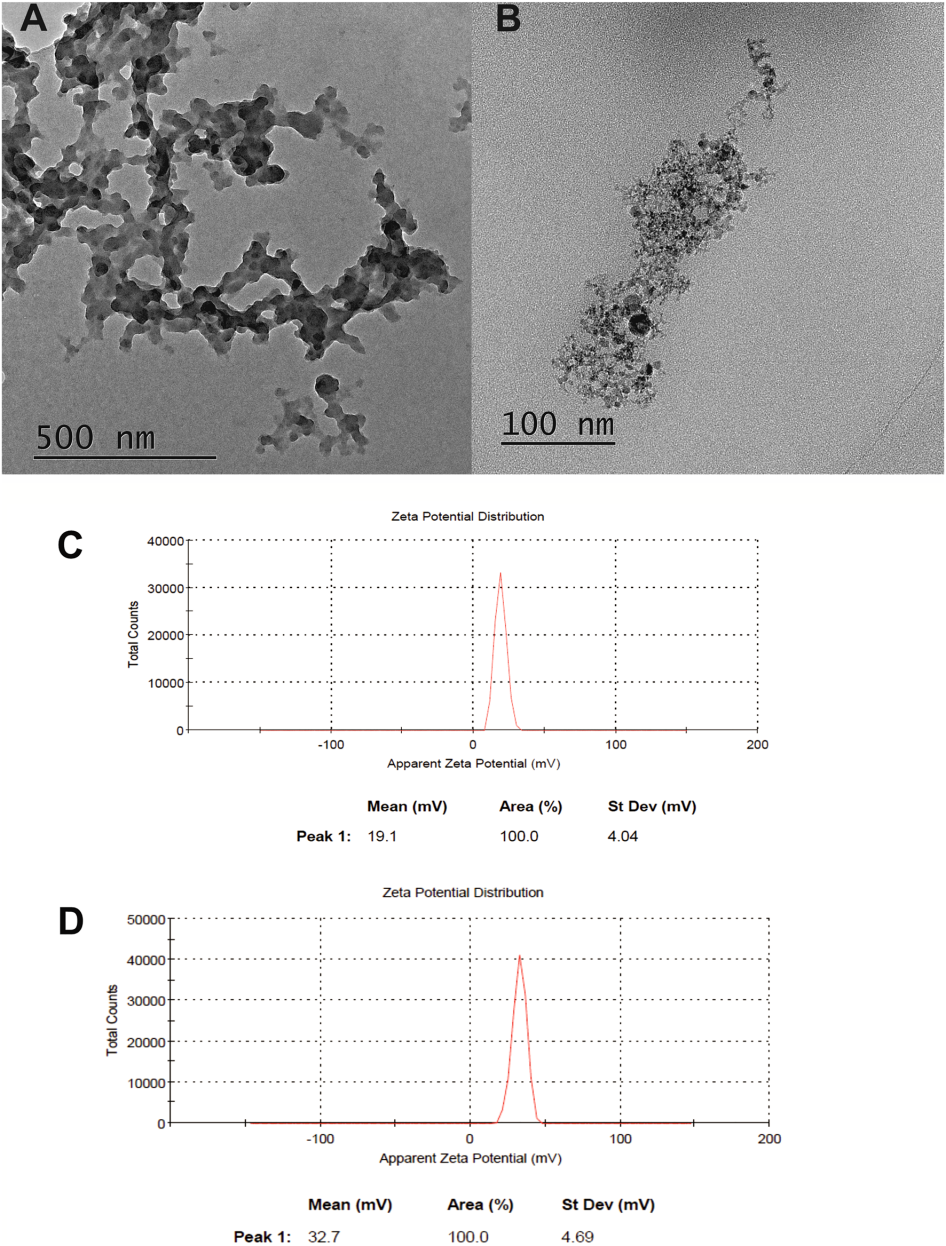
The TEM images of the siRNA-fullerene complexes that were measured for (**A**) HexakisaminoC_60_ and (**B**) JK39 carbon nanomaterials; (**C,D**) zeta potentials of the siRNA-fullerene complexes that were measured for C-HexakisaminoC_60_ and D-JK39 carbon nanomaterials.

Our next step was the physicochemical characterization of the fullerene-siRNA complexes. Firstly, we studied the changes in the zeta potential of the formed complexes, assuming that it would decrease due to the anionic character of the RNA phosphate groups. As is depicted in Fig. 3C, the zeta potential of the HexakisaminoC_60_-siRNA complex was + 19.1 mV (a change from + 28.6 mV), while the JK39-siRNA complex also had a higher zeta potential, measured at + 32.7 mV—a change from + 54 and + 90 mV (Fig. 3D). Simultaneously, the HexakisaminoC_60_-siRNA complex formed monodisperse aggregates (PDI = 0.29) around 361 nm, whereas the JK39-siRNA complex had a polydisperse mixture of aggregates around 110, 604, and 4230 nm with PDI > 0.6. The observations mentioned above regarding the size of the siRNA complexes were further investigated with TEM morphology measurements, which revealed that was a fluffy-like structure (Fig. 3A) and polydispersity for the JK39-siRNA complex (Fig. 3B) and an image of the HexakisaminoC_60_-siRNA complex which is presented in Fig. S8. The previously published reports by Nakamura et al. that described the TPFE–siRNA–plasma protein complex interactions revealed that it can disintegrate on a solid substrate, which suggests that it would also be unstable in vivo for releasing siRNA^11^.

**Figure 4:**
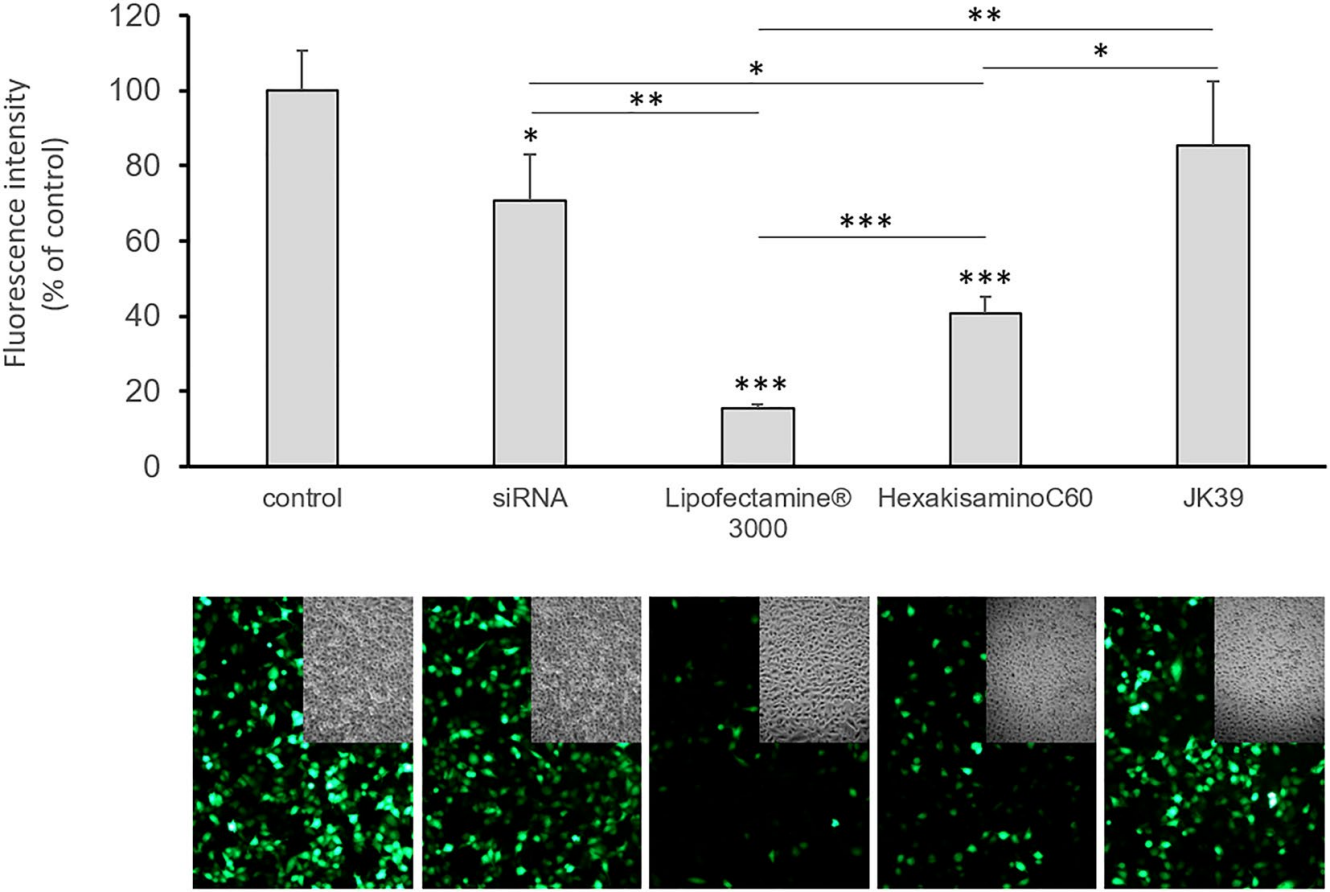
The efficiency of the siRNA transfer in vitro with the engineered fullerene nanomaterials HexakisaminoC_60_ and JK39 and the resulting EGFP silencing in a prostate cancer model (DU145 cells that had been transfected with the plasmid encoding EGFP). Each value represents the mean ± SD (n = 3). Statistical significance was determined using the t-test (*p < 0.05; **p < 0.01; ***p < 0.001).

Our final experiment was to test the siRNA transfection efficacy of the created highly water-soluble fullerene nanomaterials on prostate cancer cells that had been transfected with the EGFP-encoding plasmid and to compare their transfection properties in the presence of a serum with lipid-based Lipofectamine 3000 being used as a positive control. The results are depicted in Fig. 4, which shows a significant decrease in the fluorescence signal (to around 50%) when the cells were treated with the HexakisaminoC_60_-siRNA complex. Notably, the fullerene nanomaterial JK39 had quite a low transfections efficacy. Based on the zeta potential measurements and TEM analysis, we suspect that JK39 forms an extremely stable complex with siRNA delivering it to the cytosol but that it cannot cleave it in cellular conditions. These effects could also be caused by the d-glucosamine moiety, which has an additional nitrogen connected at C2 position of glucosamine, which could be additionally protonated (as TFA salt) and that the differences in protein corona adsorbed on the surface of fullerene nanomaterials-JK39 possess additional sugar based hydroxyl groups. This explanation complies with the higher zeta potential of sugar-based JK39 complex and thus its higher stability within cells. Moreover siRNA, when used without adding of any transfection agent, works better; it decreases the GFP fluorescence signal to 75 percent of the starting signal, which further confirms our hypothesis. An attractive option for future biological studies of the JK39 fullerene nanomaterial could be to test the photocleavage of the JK39-siRNA stable complex based on the irradiation of cells with blue/green light (glycofullerenes are photosensitizers) and the generation of ROS, which led to good results for the C_60_-Dex-NH_2_ fullerene and the upconversion nanomaterials that were used as the siRNA transfection agents^13,50^.

## Conclusions

In summary, inspired by a previously developed TPFE cationic fullerene transfection agent, we developed two cationic fullerenes HexakisaminoC_60_ and monoglucosamine JK39, which had a T_*h*_ symmetry that is characteristic for Bingel–Hirsch hexakisadducts. To the best of our knowledge, this is the first example of [60]fullerene hexakisadduct being ready to transfect siRNA. We postulate that the inactivity of JK39 fullerene in transfection experiments is caused by its high initial zeta potential and polydispersity. Future biological experiments should also determine how JK39 protects siRNA against enzymatic degradation, mainly by analyzing the adsorbed serum proteins (protein corona) on its surface. The cationic water-soluble fullerene nanomaterials to which adding interesting groups and tags (as sugars, azides or triple bonds for biorthogonal chemistry) can be attached, might hold considerable promise for in vivo siRNA delivery in the future.

## Supplementary Information

Supplementary Information.
