# Author Correction: Towards water-soluble [60]fullerenes for the delivery of siRNA in a prostate cancer model

**DOI:** 10.1038/s41598-022-21627-0

**Published:** 2022-10-19

**Authors:** Julia Korzuch, Monika Rak, Katarzyna Balin, Maciej Zubko, Olga Głowacka, Mateusz Dulski, Robert Musioł, Zbigniew Madeja, Maciej Serda

**Affiliations:** 1 Institute of Chemistry, University of Silesia in Katowice, 40-006 Katowice, Poland; 2 Faculty of Biochemistry, Biophysics and Biotechnology, Jagiellonian University, 30-387 Kraków, Poland; 3 Institute of Physics and Silesian Center for Education and Interdisciplinary Research, University of Silesia in Katowice, 41-500 Chorzów, Poland; 4 Institute of Materials Engineering, University of Silesia in Katowice, 41-500 Chorzów, Poland; 5 Department of Physics, Faculty of Science, University of Hradec Králové, 500-03 Hradec Králové, Czech Republic

Correction to: *Scientific Reports* 10.1038/s41598-021-89943-5, published online 19 May 2021

The original version of this Article contained an error in the Acknowledgments section.

“This work was supported by National Science Centre (Poland) Grant OPUS (UMO-2019/33/B/NZ7/01077) awarded to Dr. Maciej Serda.”

now reads:

“This work was supported by National Science Centre (Poland) Grant OPUS (UMO-2019/35/B/NZ7/02459) awarded to Dr. Maciej Serda.”

The original Article has been corrected.

