# Supplementary material for: Towards water-soluble [60]fullerenes for the delivery of siRNA in a prostate cancer model
Source: Sci Rep. 2021 May 19;11:10565. doi: 10.1038/s41598-021-89943-5 (PMC8134426; doi:10.1038/s41598-021-89943-5)
Supplement: Supplementary file 1 — Supplementary Information. [file 41598_2021_89943_MOESM1_ESM.pdf]

TOWARDS WATER-SOLUBLE [60]FULLERENES FOR THE DELIVERY OF siRNA IN A PROSTATE CANCER MODEL

Julia Korzuch[1], Monika Rak[2], Katarzyna Balin[3], Maciej Zubko[4,5], Olga Głowacka[2] Mateusz Dulski[4], Robert Musioł[1], Zbigniew Madeja[2] and Maciej Serda[1*]

[1]*Institute of Chemistry, University of Silesia in Katowice, Katowice, 40-006, Poland*

[2]*Faculty of Biochemistry, Biophysics and Biotechnology, Jagiellonian University, Kraków, Poland*

[3]*Institute of Physics and Silesian Center for Education and Interdisciplinary Research, University of Silesia in Katowice, 75 Pułku Piechoty 1A, 41-500 Chorzów, Poland*

[4]*Institute of Materials Engineering, University of Silesia in Katowice, Chorzow, 75 Pulku Piechoty, 1A, 41-500, Poland*

[5]*University of Hradec Králové, Faculty of Science, Department of Physics, Rokitanského 62, 500-03, Hradec Králové, Czech Republic*

**Author for correspondence: Dr. Maciej Serda (maciej.serda@us.edu.pl)*

SYNTHETIC PROTOCOL FOR OBTAINING FULLERENE JK39

NMR SPECTROSCOPY

MASS SPECTROMETRY

DLS AND ZETA POTENTIAL STUDIES

TEM IMAGE OF siRNA-HEXAKISAMINOC_{60} COMPLEX

HIGH-RESOLUTION XPS PHOTOEMISSION SPECTRA OF OXYGEN AND FLUORINE

XPS-BASED CHEMICAL COMPOSITION OF FULLERENE NANOMATERIALS

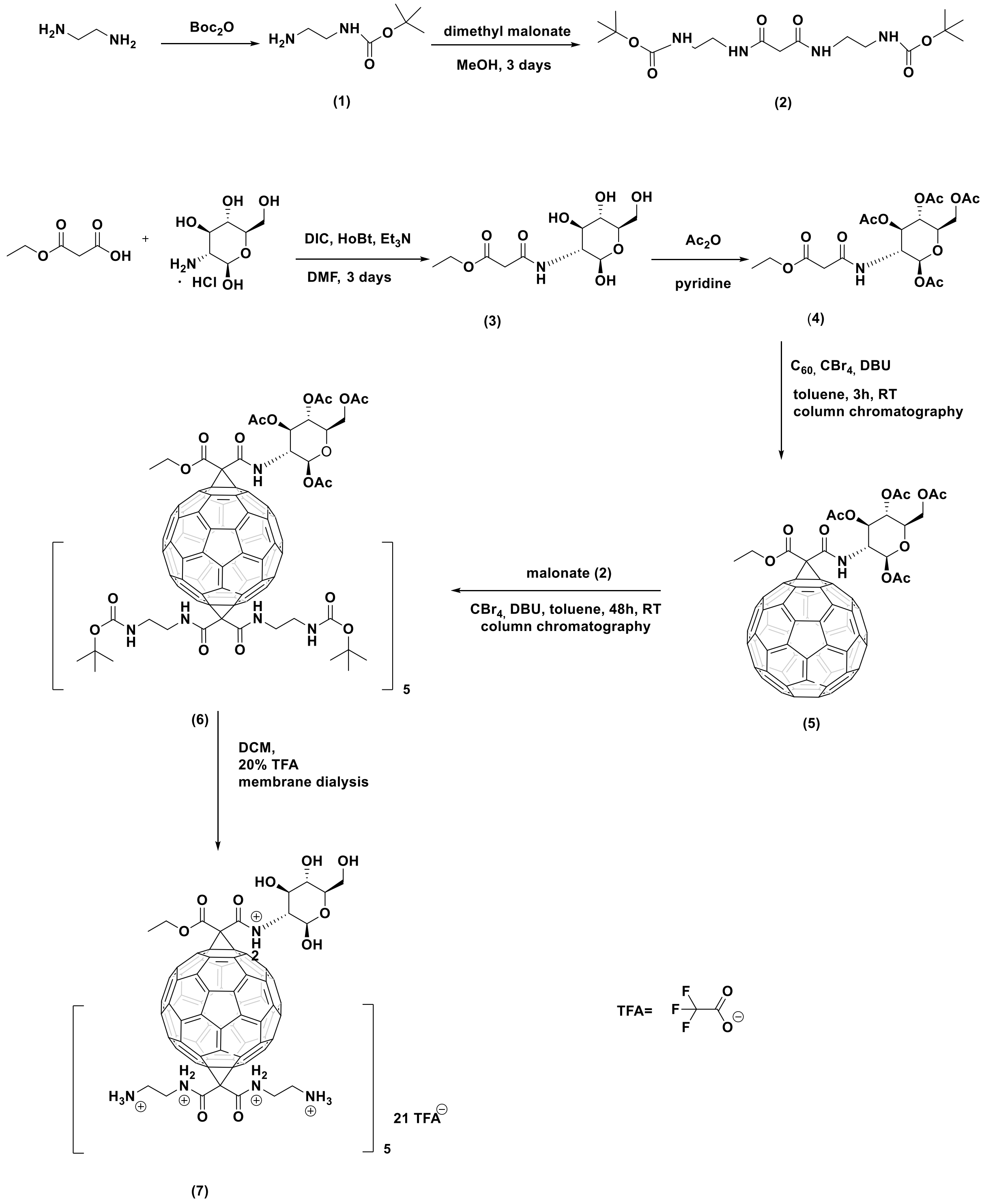

Scheme 1

Synthetic protocol for synthesis of fullerene nanomaterial **JK39**.

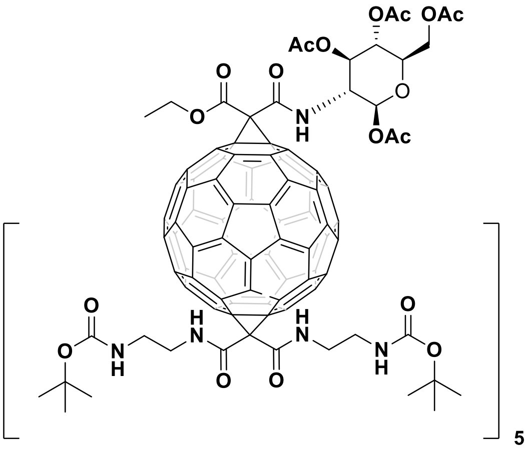

(6)

^{13}C-NMR*(d_1-$CDCl_3$,125 MHz, ppm)*: 176.98(OAc); 173.46(OAc); 171.78(OAc); 170.73(OAc); 170.02(NH-C=O); 169.11(NH-C=O); 168.66(O-C=O); 161.87(HN-C=O); 157.07(HN-COO); 146.74(C_{60}-sp^2); 140.97(C_{60}-sp^2); 90.0(glucosamine-Cα-1); 80.04(O-$\underline{C}$-$(CH_3)_3$); 70.71(glucosamine-Cα-5); 69.71(C_{60}-sp^3); 67.48(glucosamine-Cα-4); 65.29(glucosamine-Cα-3); 61.53(glucosamine-Cα-6); 60.52(-$\underline{C}H_2$-CH_3); 51.51 (glucosamine-Cα-2); 44.69($\underline{C}H_2$-NH); 43.38($\underline{C}H_2$-NH); 37.13($\underline{C}H_2$-NH); 36.19($\underline{C}H_2$-NH); 28.30(C-$(\underline{C}H_3)_3$); 20.95($\underline{C}H_3$-CO-); 20.74($\underline{C}H_3$-CO-); 20.70($\underline{C}H_3$-CO-); 20.57($\underline{C}H_3$-CO-); 13.24 (-$CH_2\underline{C}H_3$).

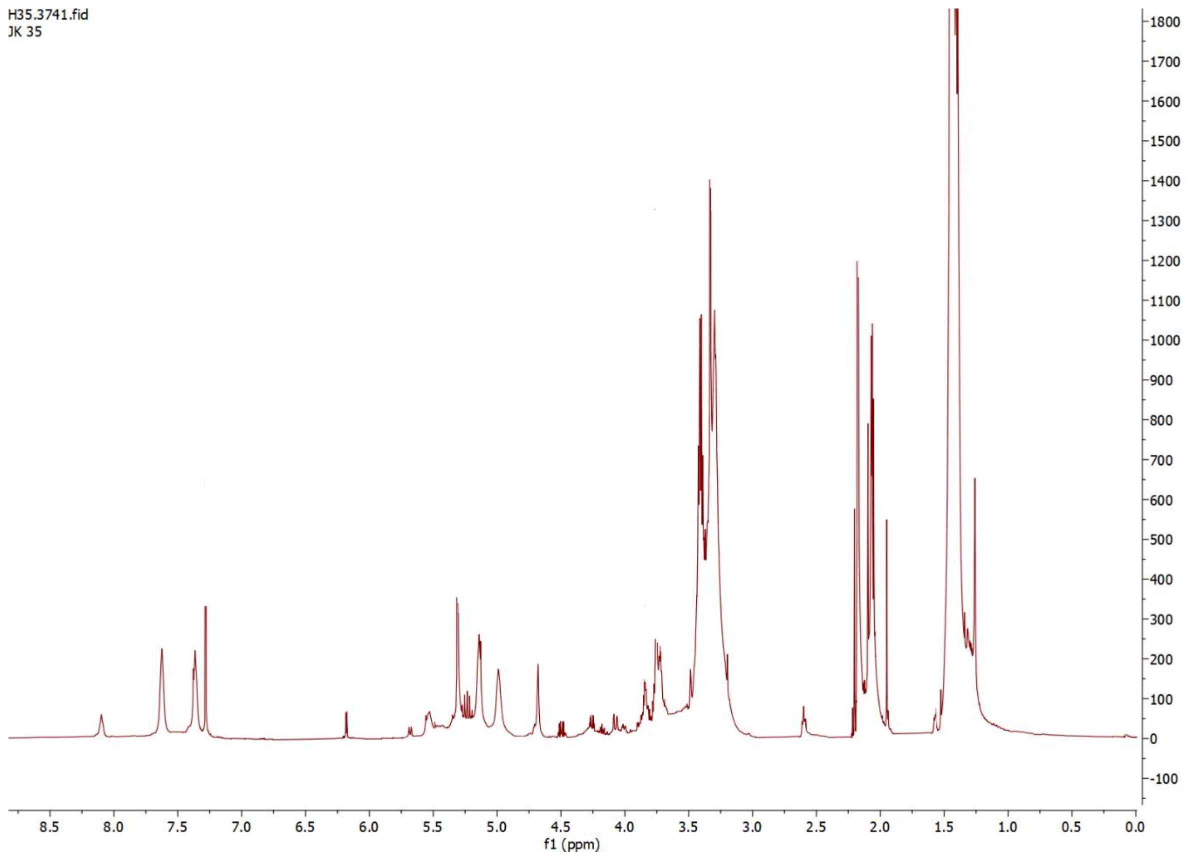

Figure S1

^{1}H-NMR spectrum of fully protected fullerene (**6**) in $CDCl_3$

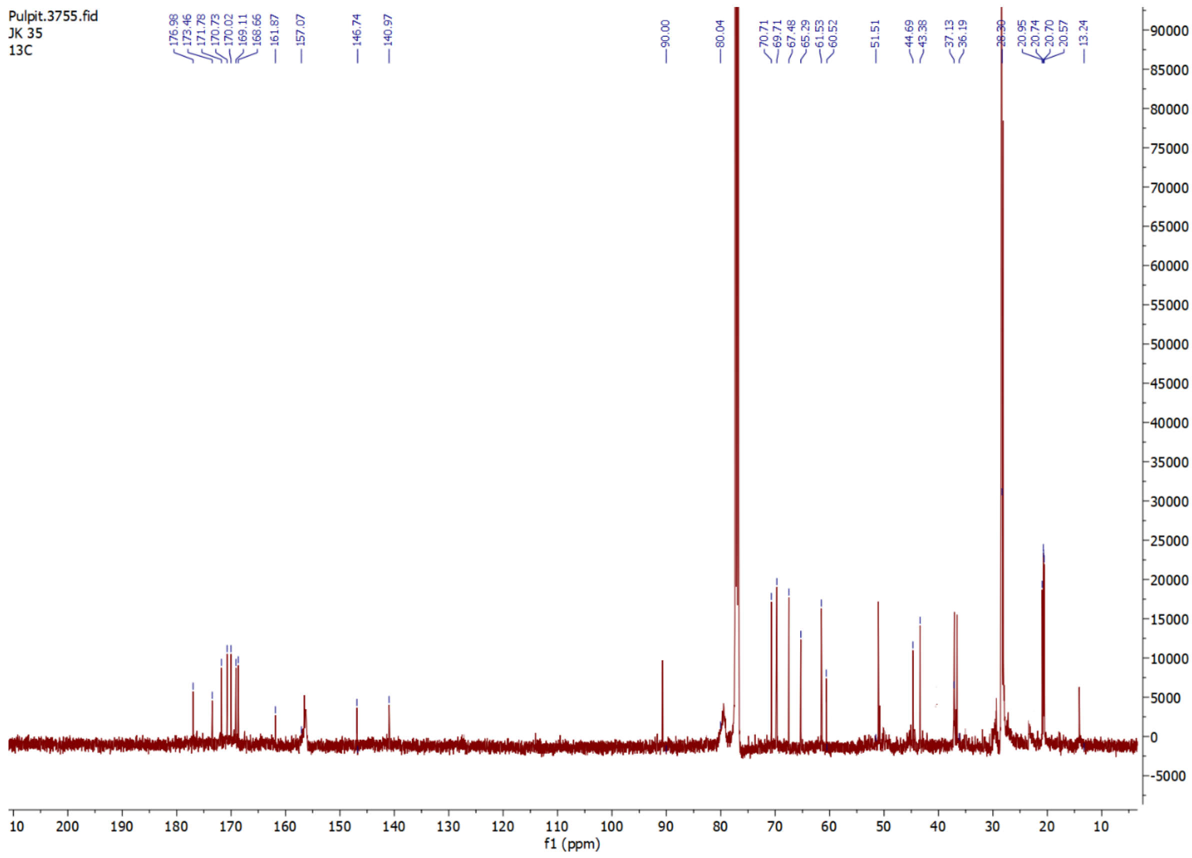

Figure S2

The ^{13}C-NMR spectrum($CDCl_3$) of protected fullerene (**6**) confirming its high symmetry (T_h) with three fullerene signals.

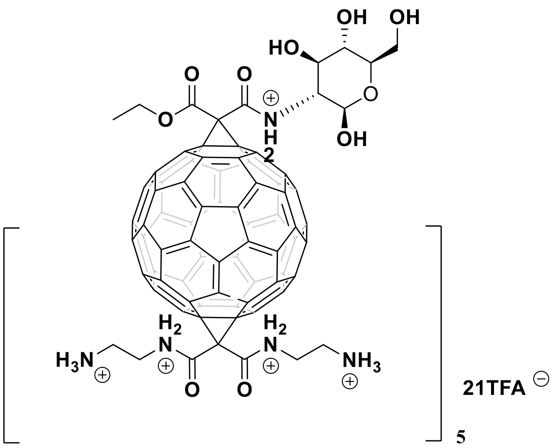

(7)

^{13}C-NMR(*d_2-D_2O,125 MHz, ppm*): 169.29(O=C-O); 163.63(O=C-NH); 162.95(O=C-NH); 162.95(q, J= 35.3 Hz, CF_3-<u>C</u>OOH); 144.79(C_{60}-sp^2); 140.45(C_{60}-sp^2); 115.6(q, J= 283 Hz, <u>C</u>F_3-COOH); 94.81($C_{1\alpha}$); 75.73($C_{5\alpha}$); 71.46($C_{3\alpha}$); 70.36($C_{4\alpha}$); 68.55(C_{60}-sp^3); 65.11(<u>C</u>H_2-CH_3); 60.51($C_{6\alpha}$); 55.56($C_{2\alpha}$); 38.39 ($NHCH_2$); 36.98 ($NHCH_2$); 13.83(CH_2-<u>C</u>H_3);

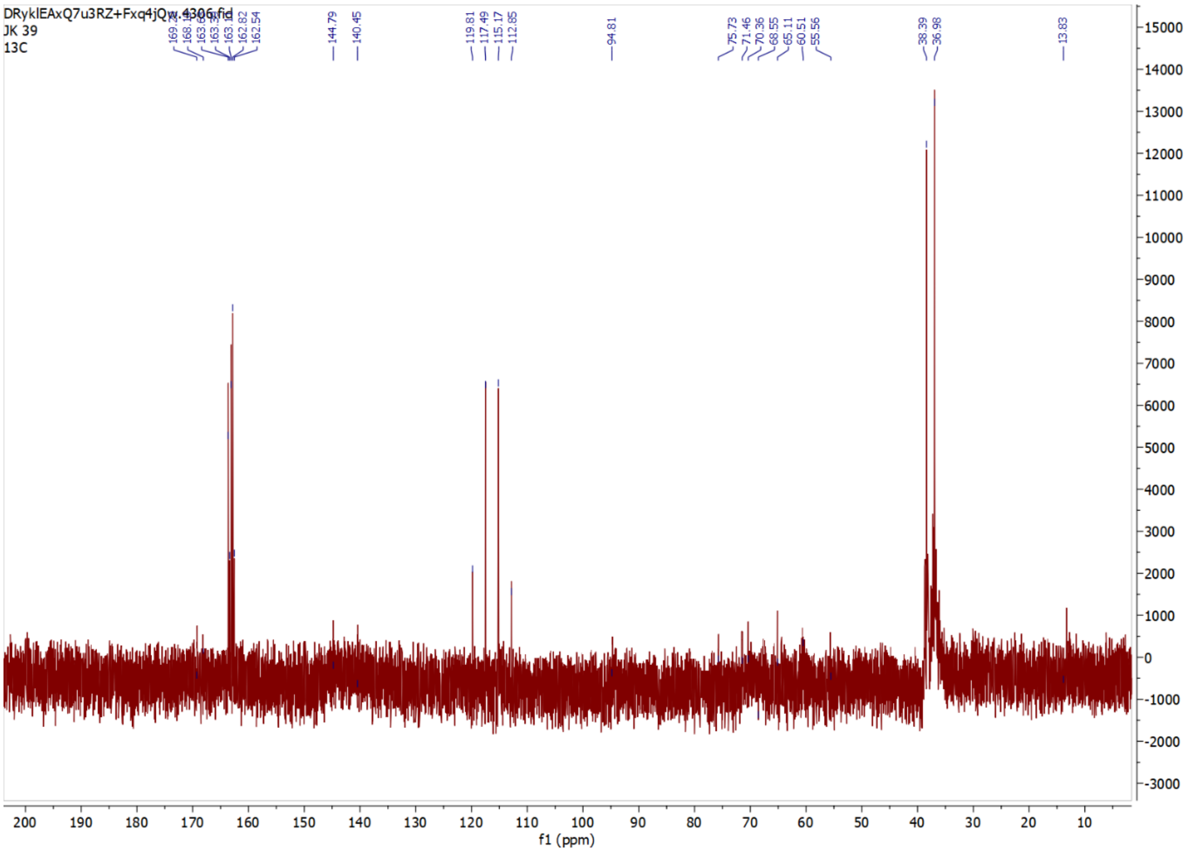

Figure S3

^{13}C-NMR spectrum of a water-soluble fullerene (**7**) confirming its high symmetry (T_h) with three fullerene signals.

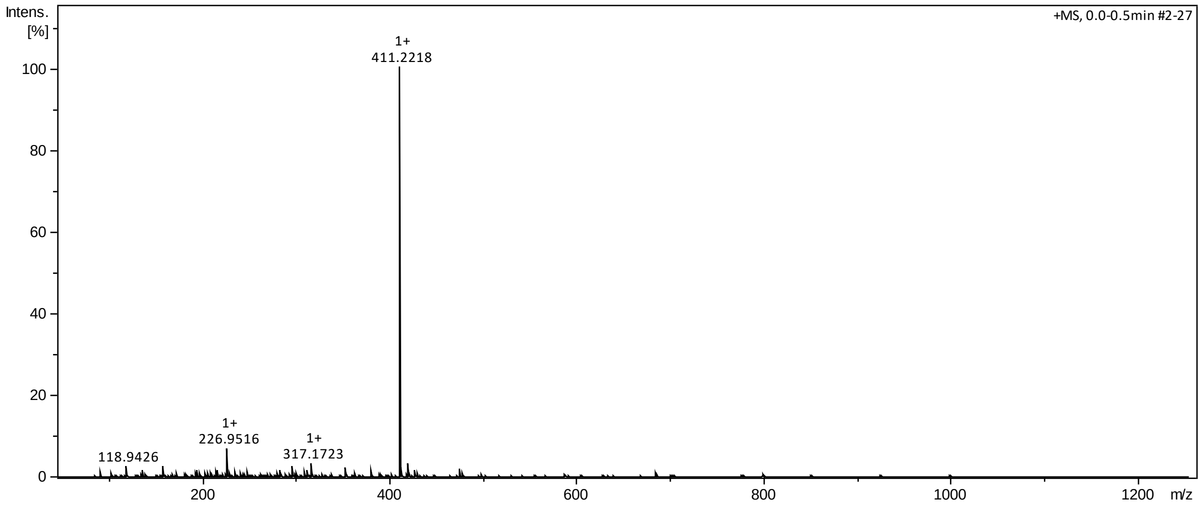

Figure S4

A high-resolution ESI-mass spectrometry of Boc-protected malonate (**2**).

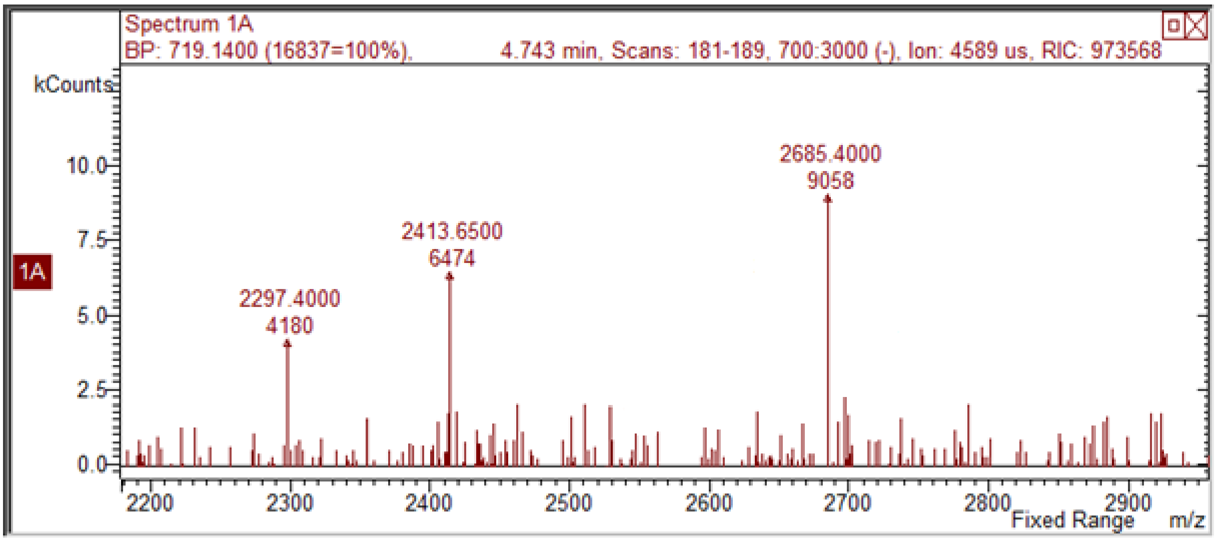

Figure S5

ESI-mass spectrum of protected hexakisadduct (**6**) in the range 2000 to 3000 Da in a positive mode (100 mV).

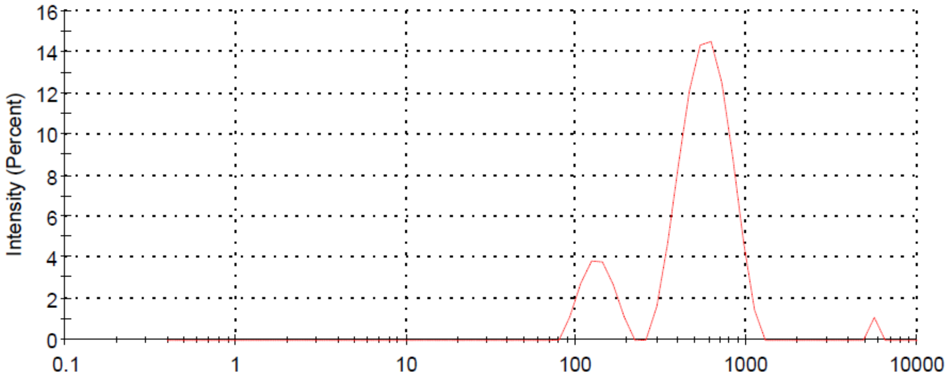

	Size (d.n...	% Intensity:	St Dev (d.n...
**Peak 1:**	598.7	83.5	180.7
**Peak 2:**	134.1	15.4	26.89
**Peak 3:**	5560	1.1	0.000

Figure S6

Size measurement of fullerene nanomaterial **JK39** by dynamic light scattering (DLS) technique.

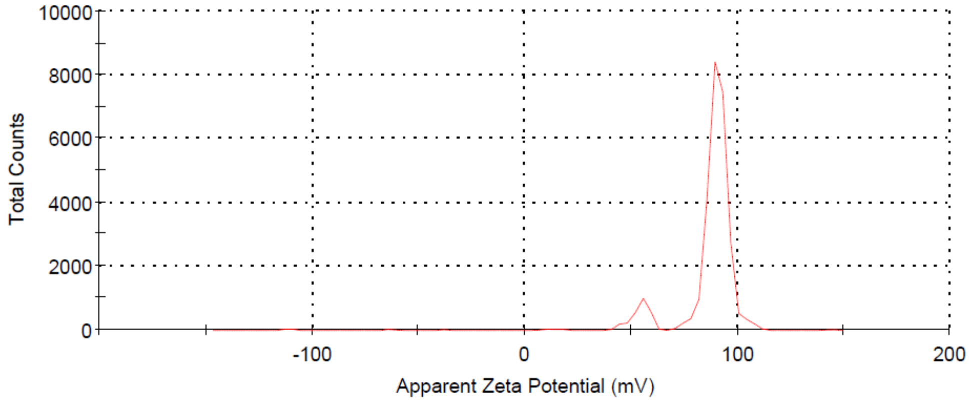

	Mean (mV)	Area (%)	St Dev (mV)
**Peak 1:**	90.5	89.9	5.19
**Peak 2:**	54.1	9.2	4.55
**Peak 3:**	13.7	0.3	3.44

Figure S7

Zeta potential measurement of fullerene nanomaterial **JK39.**

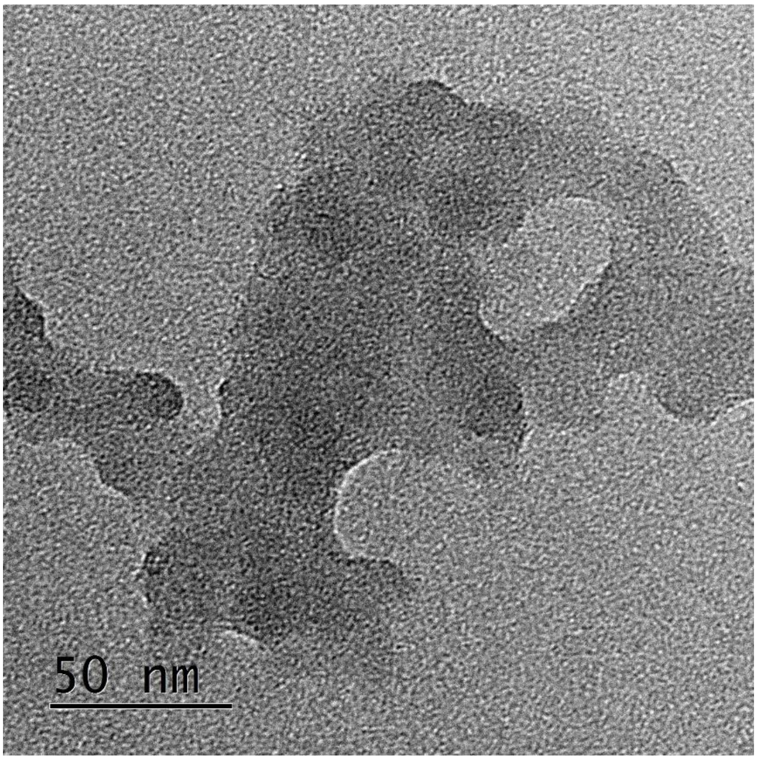

Figure S8

The TEM image of HexakisaminoC_{60}-siRNA complex.

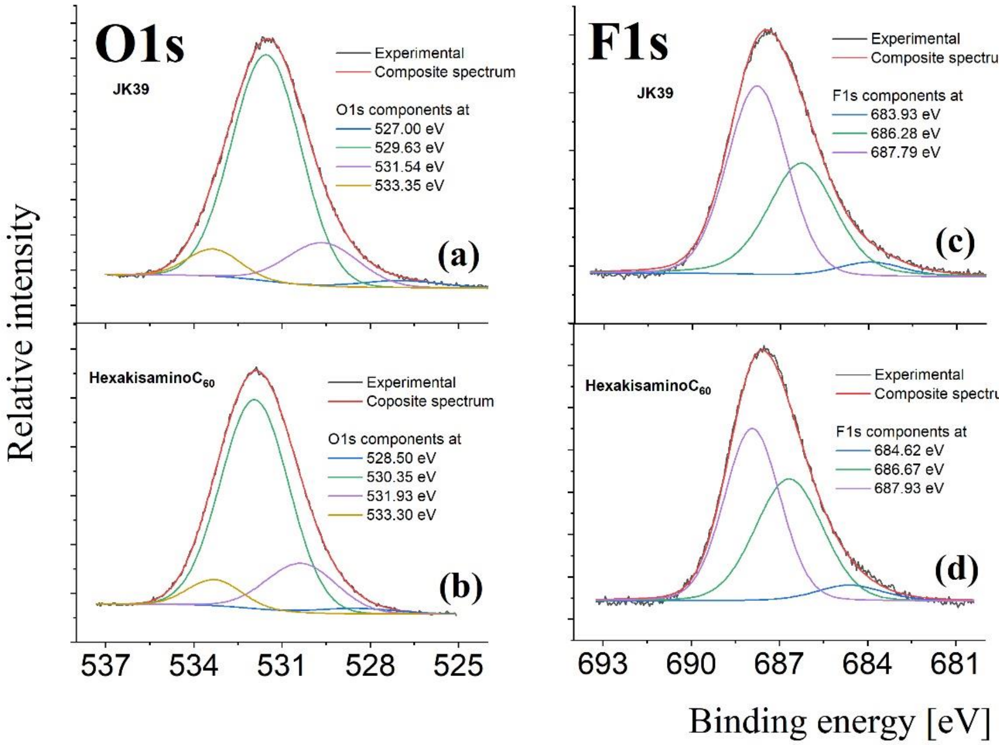

Figure S9

A high-resolution photoemission spectra of oxygen (a) JK39, (b)HexakisaminoC_{60} and fluorine (c) JK39, (d) HexakisaminoC_{60}, measured in selected fullerene nanomaterials.

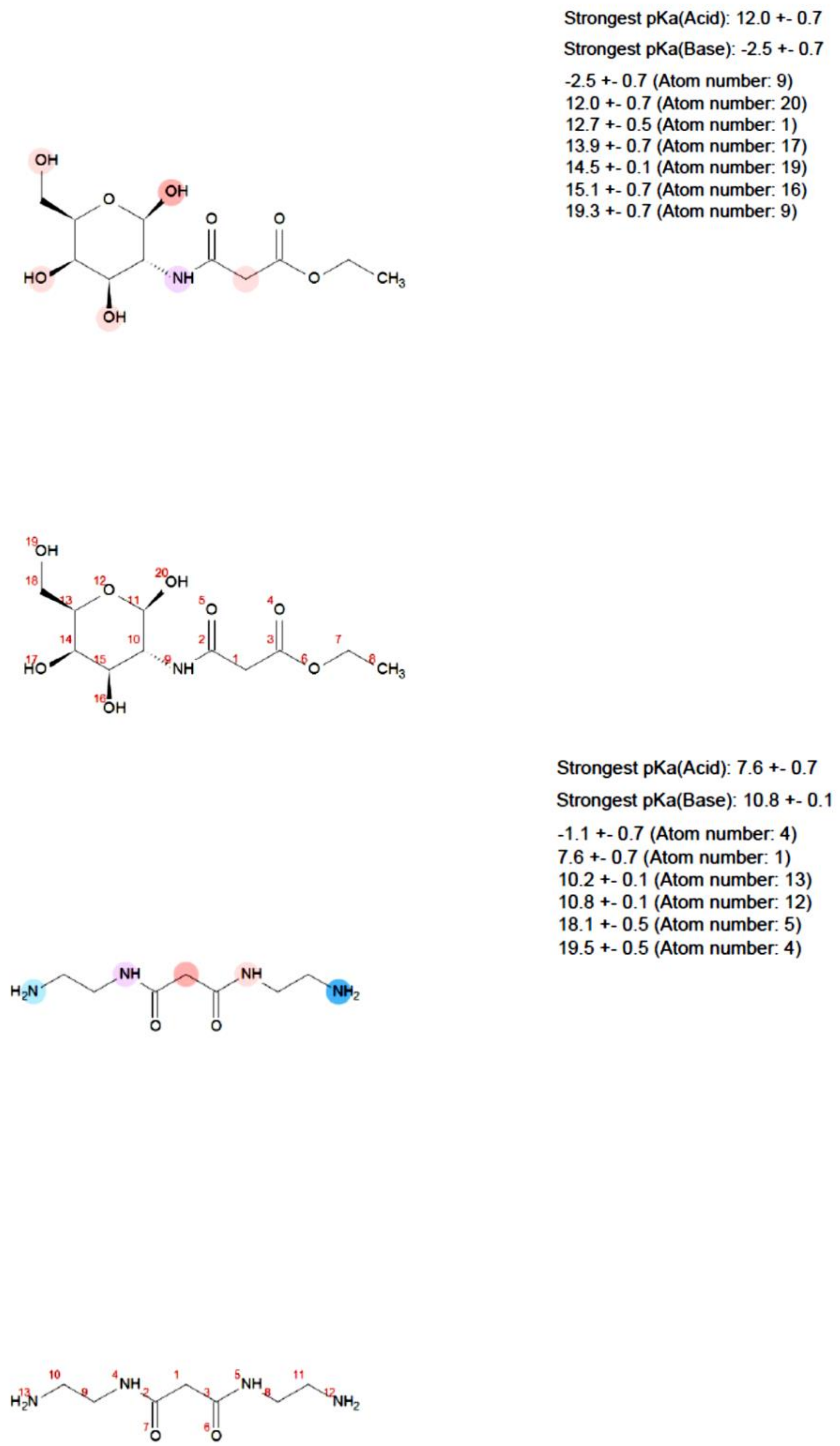

Figure S10

Calculation of all nitrogen pK_a parameters in selected malonates used for synthesis of fullerenes JK39 and HexakisaminoC$_{60}$. All calculations were performed using ACD Percepta software. The color dots represent specific order of basic groups

	JK39					HexakisaminoC$_{60}$				
Element	Total weight %	Atomic concentration of particular element	Binding energy [eV] of detected chemical states	Percentage of total amount of given element	Atomic concentration of detected chemical state	Total weight %	Atomic concentration of particular element	Binding energy [eV] of detected chemical states	Percentage of total amount of given element	Atomic concentration of detected chemical state
C	55.18	62.93	282.42	5.53	3.48	57.83	66.11	282.58	4.52	2.99
			284.82	83.08	52.28			284.82	84.03	55.55
			287.12	5.82	3.66			287.10	7.62	5.04
			288.18	5.57	3.51			288.51	3.82	2.53
O	22.28	19.08	527.00	2.74	0.52	23.34	20.03	528.50	2.31	0.46
			529.63	14.89	2.84			530.35	17.62	3.53
			531.54	75.48	14.40			531.93	72.97	14.62
			533.35	6.89	1.31			533.30	7.1	1.42
N	10.23	10.00	395.70	3.13	0.31	6.65	6.52	396.26	1.68	0.11
			398.00	22.00	2.20			398.00	13.63	0.89
			399.60	72.12	7.21			399.60	76.42	4.98
			401.46	2.74	0.27			401.70	8.27	0.54
F	9.50	6.85	683.93	5.27	0.36	6.97	5.03	684.62	6.25	0.31
			686.28	39.94	2.74			686.67	43.24	2.17
			687.79	54.79	3.75			687.93	50.51	2.54
Si	1.81	0.88	-	-	-	2.74	1.34	-	-	-
S	-	-	-	-	-	1.61	0.69	-	-	-
Na	0.11	0.07	-	-	-	0.20	0.12	-	-	-
Cl	0.21	0.08	-	-	-	0.25	0.10	-	-	-
Br	0.68	0.12	-	-	-	0.41	0.07	-	-	-

Table S1

Chemical composition and atomic and weight concentrations determined from XPS measurements. High-resolution photoemission spectra were not measured for the Si, S, Na, Cl, and Br.
